# Shedding Light on the Antimicrobial Peptide Arsenal of Terrestrial Isopods: Focus on Armadillidins, a New Crustacean AMP Family

**DOI:** 10.3390/genes11010093

**Published:** 2020-01-14

**Authors:** Thomas Becking, Carine Delaunay, Richard Cordaux, Jean-Marc Berjeaud, Christine Braquart-Varnier, Julien Verdon

**Affiliations:** Laboratoire Ecologie et Biologie des Interactions, UMR CNRS 7267, Université de Poitiers, 86073 Poitiers, France; thomas.becking@univ-poitiers.fr (T.B.); carine.delaunay@univ-poitiers.fr (C.D.); richard.cordaux@univ-poitiers.fr (R.C.); jean-marc.berjeaud@univ-poitiers.fr (J.-M.B.)

**Keywords:** crustacea, malacostraca, terrestrial isopods, innate immunity, antimicrobial peptides, glycine-rich peptides, transcriptomics

## Abstract

In crustaceans, antimicrobial peptides (AMPs) are clustered into four major groups according to their amino acid composition and structure: (1) single-domain peptides containing cysteine residues such as anti-lipopolysaccharide-factor (ALF), (2) multi-domain or chimeric AMPs such as crustins, (3) non-conventional AMPs, and (4) linear single-domain AMPs. The majority of AMPs has been described in commercially exploited crustaceans, particularly decapods living in aquatic environments (crab, shrimp, lobster, and crayfish). Here, we aimed at establishing the AMPs repertoire of terrestrial isopods (Oniscidea), an original suborder of crustaceans adapted to life outside of the aquatic environment. Using transcriptomic data from 21 species, we identified 110 ALF and 73 crustin sequences. We also characterized the full-length sequence of armadillidins from 17 species, similar to the AMP previously described in the terrestrial isopod *Armadillidium vulgare*. Furthermore, we tested the antimicrobial activity of three armadillidin peptides characterized from three distantly related species. This analysis revealed similar activity spectra against pathogens, despite extensive structural variation among the tested peptides. In addition to conventional crustacean AMPs, our work highlights armadillidins as a new and independent family of AMPs specific to the Oniscidea, thus opening new perspectives concerning the study of the immune system of terrestrial isopods.

## 1. Introduction

Crustaceans form a large, ancient and extremely diverse animal group. After insects, they are by far the most numerous and widespread animals on Earth. Crustaceans are primarily marine organisms and they constitute a large proportion of the biomass of oceans [1], but there are also freshwater, semi-terrestrial and terrestrial species. Over their long evolutionary history, crustaceans have been facing a wide variety of integrity challenges because their natural habitat is generally overloaded with infectious organisms, such as viruses, bacteria, fungi and other parasites [2]. Their evolutionary success confirms the effective strategies they use to fight against any kind of disease-causing agents and parasites present in their environment.

Since pathogens are a threat to their survival, crustaceans have evolved efficient mechanisms based on two specific and complementary immune responses that may contribute to the elimination of invaders [3]: (i) the cellular response, resulting in phagocytosis of small particles and encapsulation of larger ones, and (ii) the humoral response involving the synthesis and the release of several immune proteins into the hemolymph, such as clotting enzymes, phenoloxidase cascade effectors, and antimicrobial peptides (AMPs).

AMPs, also known as host defense peptides (HDPs), are widespread in living organisms ranging from bacteria to humans, and are an evolutionarily conserved component of the innate immune system shared by all classes of life [4]. AMPs can inhibit or kill a broad spectrum of bacteria, fungi, yeast, protozoa and viruses at micromolar concentrations. All AMPs share common features: (i) small size acids in length (ii) cationic and hydrophobic properties, and (iii) amphipathic structure [5]. Due to the extreme diversity in their primary structure, AMPs are commonly classified into three main classes according to their spatial structure and/or amino acid composition: (a) linear α-helical peptides, (b) linear peptides (enriched with amino acid residues such as Arg, Gly, Pro or Trp), and (c) cysteine-containing peptides (stabilized by intermolecular disulfide bonds) [4].

In crustaceans, there are 15 distinct AMP families or simple peptides sharing common molecular characteristics with currently known AMP families, published so far in the literature. Some AMP families are found in all crustaceans studied, such as the anti-lipopolysaccharide-factor (ALF) [2,6], while others are specific to given lineages, such as the penaeidins restricted to penaeid shrimp. With only a few exceptions, all these AMP families have antimicrobial activity against a number of specific microorganisms [2]. Based on the structure and amino acid composition, crustacean AMP families can be clustered into four main groups [2,6]: (i) single-domain linear α-helical AMPs and peptides enriched in specific amino acids, (ii) single-domain peptides containing cysteine residues engaged in disulfide bonds such as ALF, (iii) multi-domain or chimeric AMP-like crustins, which are shared by several crustacean species, and (iv) unconventional AMPs including multifunctional proteins or protein-derived fragments that exhibit antimicrobial functions.

Given the ecological and economic significance of aquatic crustaceans, almost all of the best characterized AMPs were characterized from species of commercial interest, such as marine decapods (shrimp, lobster, and crab) [6]. However, the order Decapoda is far from representing the entire breadth of diversity of crustaceans. To broaden our knowledge of the diversity, structure, function and evolution of crustacean AMPs beyond decapods, we characterized the AMP repertoire of terrestrial isopods. Belonging to super-order Peracarida, terrestrial isopods are the most successful colonizers of terrestrial habitats among crustaceans [7]. They constitute an important component of soil fauna, in which they act as scavengers. As such, they are in permanent contact with abundant and diverse communities of microorganisms [7,8,9]. It is therefore conceivable that these crustaceans are equipped with a specific arsenal of AMPs.

In this study, we aimed at deeply characterizing the immune repertoire of terrestrial isopods, with particular focus on three AMP families: ALFs, crustins and armadillidins. ALFs and crustins constitute the two major AMP families known in crustaceans and they are well-conserved in malacostracans in general, not just decapods [2]. Armadillidins are glycine-rich AMPs originally identified from the terrestrial isopod *Armadillidium vulgare* [10,11]. To date, only two armadillidin isoforms, armadillidin H and armadillidin Q, have been described [11]. Using the transcriptomes of 21 terrestrial isopod species generated in a previous study [12], we identified, characterized, and compared ALFs and crustins. We also surveyed these transcriptomes for the presence of armadillidins and identified 16 new armadillidin sequences with a characteristic molecular signature, i.e., a high presence of a G-rich motif (GGGX). A search extended to other crustaceans available in open access databases has not enabled the presence of armadillidins to be identified, suggesting that armadillidins are specific to terrestrial isopods. Determination of the antimicrobial activity of three different armadillidins revealed that they have a broad-spectrum antimicrobial activity, similar but not identical to the one previously described for armadillidin H and armadillidin Q [11]. Altogether, these findings suggest that armadillidins constitute a new crustacean AMP family, which evolved more than 100 million years ago, possibly in connection with adaptation to terrestriality.

## 2. Materials and Methods

### 2.1. Transcriptome Datasets and Preparation of Query Sets

We used full transcriptome datasets from 21 terrestrial isopod species generated in a previous study [12]. We first generated a non-redundant dataset using CD-HIT (version 4.6, https://github.com/weizhongli/cdhit). All contigs with hits ≥ 95% nucleotide identity were collapsed to remove potential splice variants from the analyses. Terrestrial isopod transcripts were translated in silico according to the longest open-reading frames into amino acid sequences, as described in earlier work [12].

For query sequences used in homology searches, we used the ALF isoform 2 from the black tiger shrimp *Penaeus monodon* (accession number: ABP73291), crustin 1 from the Japanese spiny lobster *Panulirus japonicus* (accession number: ACU25382), and armadillidin H isolated from the woodlouse *Armadillidium vulgare* (accession number: AAU14168).

### 2.2. In Silico Characterization and Phylogenetic Analyses of Crustins and ALFs

ALF and crustin transcripts from terrestrial isopod transcriptomes were characterized using a reciprocal BLAST approach. ALF and crustin queries were first aligned against transcriptome datasets using BlastP [13], with an E-value threshold set at 10^−10^. To confirm the identification of these sequences as ALF or crustin, they were then compared to the non-redundant (NR) database of NCBI (version September 2017) using BlastP [13]. Conserved domains of the terrestrial isopod hits were annotated using the Batch CD-Search Tool by NCBI (https://www.ncbi.nlm.nih.gov/Structure/bwrpsb/bwrpsb.cgi). Hits without essential domains belonging to ALF (DUF3254 domain, Pfam: PF11630) or crustin (WAP domain, Pfam: PF00095) were discarded. Indeed, Decapoda ALF sequences are characterized by a large mature peptide (11 kDa) containing a highly hydrophobic N-terminal region and two cysteine residues [2,14,15,16]. All ALFs sequences belong to the DUF3254 superfamily, characterized by the presence of the DUF3254 protein domain [17,18]. All crustin precursors have a leader sequence at the N-terminal region and a mature multi-domain cationic AMP (7–14 kDa) characterized by the always present WAP (whey acidic protein) domain at the C-terminus [2,14,15,19,20].

Multiple alignments of ALF and crustins were generated with the Geneious aligner (version 7.0.6) [21] using default parameters. Alignments were then trimmed with GBLOCKS (version 0.91b) [22] to remove ambiguously aligned regions. To maximize the resulting alignment length, we used the less stringent options to obtain 112 and 98 amino acid alignment lengths for ALF and crustin sequences, respectively (fasta files are available in Supplementary Files S1 and S2). To determine the best-fit model of nucleotide substitution, we used Prottest (version 3.4) [23]. The best substitution model was LG + G for ALF and JTT + I + G for crustin, according to all information criteria. Maximum-likelihood analyses were performed independently on each protein alignment using RAxML (version 7.4.6) [24] with 100 independent replicates, followed by 1000 replicates of bootstrap resampling. Bootstrap values were subsequently mapped onto the optimal consensus tree obtained from the 100 independent searches. Only nodes having a bootstrap value ≥ 50% were considered. Final tree files are available in newick format in Supplementary Files S3 and S4. Graphical representations of Newick trees were generated using iTOL [25]. Finally, peptide signals of ALFs and crustins were predicted and characterized using the Phobius web-server [26].

### 2.3. Identification and Characterization of Armadillidins

The armadillidin H query was aligned against transcriptome datasets using BlastP [13] with an E-value threshold set at 10^−10^. All armadillidin sequences identified in silico were confirmed by PCR and Sanger sequencing using the degenerated primers previously designed by Herbinière and colleagues [10]. To obtain full-length armadillidin sequences in *Armadillo officinalis* and to include the armadillidin sequence of *Porcellio dilatatus* (not included in the species set of Becking et al. [12]), we designed PCR primers and Sanger-sequenced resulting amplification products. PCRs were performed as follows: 3 min at 94 °C for the initial denaturing step, followed by 35 cycles of 30 s at 94 °C; 30 s at 50 °C or 55 °C (depending on the melting temperature of the primers); and 1 min at 72 °C. The final elongation step was 10 min at 72 °C. Primer sequences, PCR product sizes, and melting temperatures are provided in Supplementary Table S1. Purified PCR products were sequenced on an ABI PRISM 3130xl automated sequencer (Applied Biosystems, Foster City, CA, USA). Peptide signals of armadillidins were predicted and characterized using the Phobius web-server [26].

To investigate the evolution of the full mature form of armadillidins during terrestrial isopod diversification, full-length sequences (i.e., from first methionin to stop codon) were mapped onto the phylogeny of terrestrial isopods previously published [12].

### 2.4. Bacterial Growth Inhibition Assays

Synthetic native peptides armadillidin CE (molecular weight of 5150.5 g/mol) and armadillidin PP (molecular weight of 5307.6 g/mol), with the respective amino acid sequences (without C-terminal amidation) TPGRPYYGGGYNGGYRGGYRRGGGFYGGGRFYGGGEGYRGGYYRGYRG and SYGRGSYGGGSIGRGSFGHGGGSFGRGGGRFGHGGGRFGGIGGGGRYGGGHIGGYRG were purchased from ProteoGenix Corporation (Schiltigheim, France). Stock solutions were prepared in 8% acetonitrile at a final concentration of 1.8 mM and stored at −80 °C. Working solutions were prepared by dilution in sterile water. All other reagents were purchased from Sigma-Aldrich (Saint-Louis, MO, USA) unless stated otherwise.

Minimum inhibitory concentrations (MIC) of both peptides were measured towards various bacterial strains as previously described [11]. MIC was defined as the lowest concentration of peptide that totally inhibits the visible growth of a selected bacterial strain after a 24-h incubation period. Bacterial strains used in this study are listed in Table 1. Bacteria were grown for 24 h on either nutrient agar plates or broth under shaking (200 rpm), at 28 °C or 37 °C depending on the tested strain.

## 3. Results and Discussion

### 3.1. Description of the ALF and Crustin Repertoire of Terrestrial Isopods

**Figure 1:**
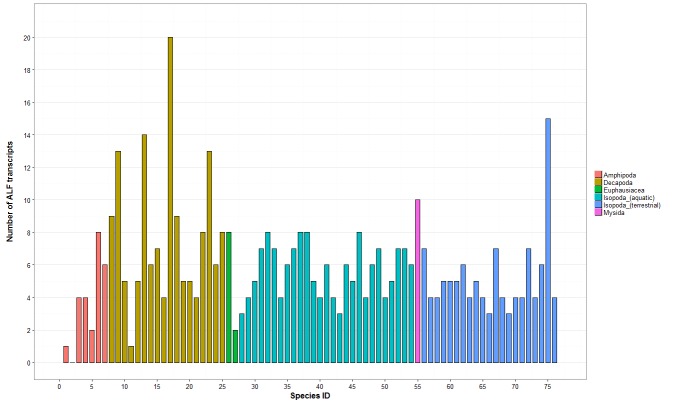
Amount of anti-lipopolysaccharide- factor (ALF) transcripts identified in 76 malacostracans. The *y*-axis represents the total number of transcripts. ALF transcripts identified in Amphipoda, Decapoda, Euphausiacea, aquatic Isopoda, and Mysidia are from [27]. ALF transcripts from terrestrial Isopoda were identified in this study. Each species is represented by a number on the *x*-axis and the full list of species is available in Supplementary Table S2.

**Figure 2:**
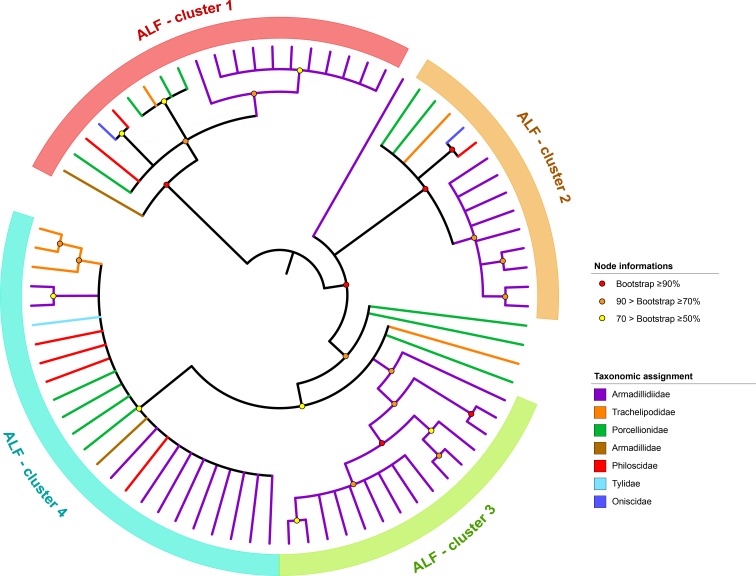
Phylogenetic analysis of ALF transcripts identified in 21 terrestrial isopod transcriptomes. The phylogenetic tree was inferred by maximum-likelihood, based on the DUF3254 domain, corresponding to a 112 amino acid alignment of 78 sequences. Only nodes having a bootstrap value ≥ 50% were considered.

Our analysis identified a total of 110 AFLs in the transcriptomes of the 21 terrestrial isopod species (Supplementary Table S2). We identified 5.2 (±2.5) AFL transcripts per terrestrial isopod species on average, which is quite similar to the average number of ALF transcripts previously described in several crustacean species (6.2, ±3.3) (Figure 1). The highest number of ALFs (15 transcripts) was identified in *Trachelipus rathkei* (Figure 1 and Supplementary Table S2). Sequence analysis of terrestrial isopod ALFs indicated that they have two conserved cysteine residues suggestive of disulfite bridge formation [27] (Supplementary File S1), as previously described for other malacostracan ALFs [27]. The region located between these cysteine residues is considered as a LPS-binding domain formed with positively charged amino acids [27]. As this domain is also present in other malacostracan ALFs [27], it suggests LPS-binding conservation across the whole gene family. To investigate ALF evolutionary history in terrestrial isopods, we performed a phylogenetic analysis using the amino acid sequences of the 78 ALF transcripts ≥ 100 amino acids in length. Results showed that the 78 ALFs are grouped in four major clusters (Figure 2). Clusters 1, 2 and 4 contain sequences from multiple terrestrial isopod families whereas cluster 3 is composed of ALFs belonging solely to the Armadillidiidae family (Figure 2). The phylogenetic distribution of ALF sequences suggests that the four clusters were probably present in the common ancestor of all terrestrial isopods, more than 110 My ago (according to [12]). We also noticed that the signal peptide of ALFs from clusters 1 and 4 are highly similar, as 18/24 amino acids of the peptide signal consensus sequence share similar physico-chemical properties (Supplementary Figure S1). These data suggest the existence of a similar secretory pathway for these two ALF subfamilies.

**Figure 3:**
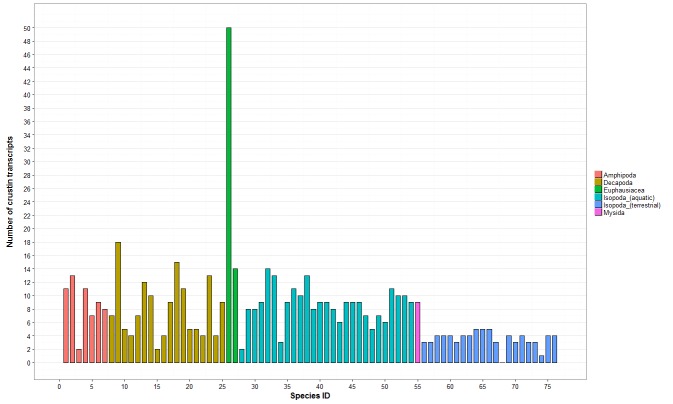
Amount of crustin transcripts identified in 76 malacostracans. The *y*-axis represents the total number of transcripts. Crustin transcripts identified in Amphipoda, Decapoda, Euphausiacea, aquatic Isopoda, and Mysidia are from [27]. Crustin transcripts from terrestrial Isopoda were identified in this study. Each species is represented by a number on the *x*-axis and the full list of species is available in Supplementary Table S2.

**Figure 4:**
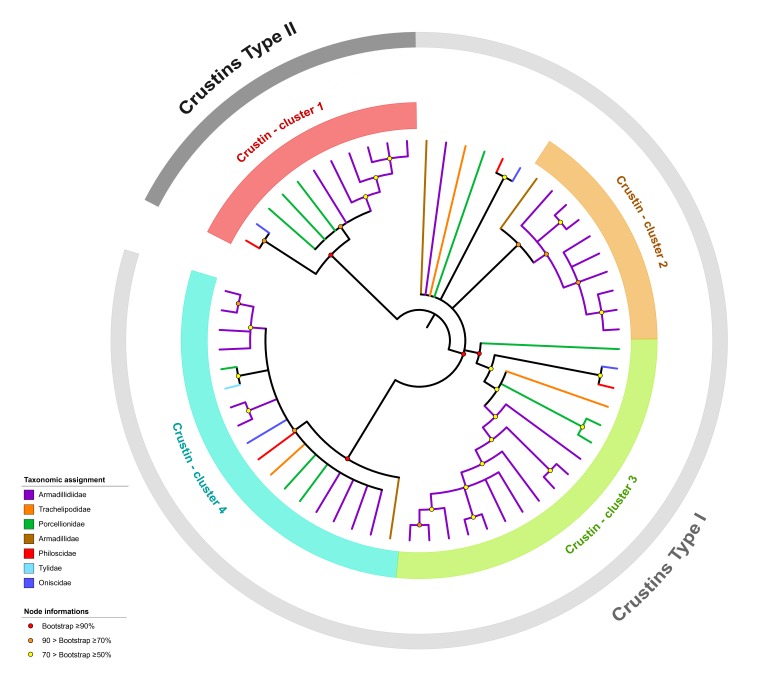
Phylogenetic analysis of crustin transcripts identified in terrestrial isopod transcriptomes. The phylogenetic tree was inferred by maximum-likelihood, based on the WAP domain, corresponding to a 98 amino acid alignment of 63 sequences. Only nodes having a bootstrap value ≥ 50% were considered.

Our analysis also identified a total of 73 crustins in the transcriptomes of 20 of the 21 terrestrial isopod species (no crustin was detected in *Porcellio dispar*; Supplementary Table S2). We identified 3.5 (±1.2) crustin transcripts per terrestrial isopod species on average, which is almost three times lower than the average amount of crustin transcripts previously described in other crustacean species (9.2, ±6.6; Figure 3). The highest number of crustins in terrestrial isopods (five transcripts) was identified in *Armadillidium tunisiense*, *Eluma purpurascens* and *Porcellio laevis* (Figure 3 and Supplementary Table S2). The crustin WAP domains from terrestrial isopods contained 12 conserved cysteines residues, as previously described for other malacostracans [27]. To investigate crustin evolutionary history in terrestrial isopods, we performed a phylogenetic analysis using the amino acid sequences of the 63 crustin transcripts ≥ 90 amino acids in length. Results showed that the 63 crustins are grouped in four major clusters (Figure 4). All clusters contain sequences identified from multiple terrestrial isopod families, suggesting that they are widespread among terrestrial isopod species. As for ALF sequences, the phylogenetic distribution of crustin sequences suggests that the four clusters were probably present in the common ancestor of all terrestrial isopods, more than 110 My ago. Following the classification proposed by Valgas-Albores and Martínez-Porchas [28] based on the size of the G-rich region, the proportion of G content and the presence of repetitive tetrapeptides, crustin transcripts from cluster 1 can be considered as type II crustins and sequences belonging to clusters 2, 3, and 4 can be considered as type I crustins (Table 1). Another classification proposed by Tassanakajon and colleagues [14] based on the presence of a cysteine-rich domain, a glycine-rich hydrophobic domain, a proline and arginine rich region, an aromatic amino acid-rich domain, and on the number of the WAP domain. It allows us to classify crustin transcripts from cluster 1 as type II crustins and sequences belonging to clusters 2, 3, and 4 as type I crustins (Table 1). Usually, type I crustins are only active against Gram-positive bacteria and are found in several malacostracan species such as crabs, crayfishes, and lobsters [14,15]. However, little is still known about the biological activities and molecular functions of type I crustins [14,15]. Type II crustins are the most abundant crustins identified in malacostracans. They exhibit a highly variable mixture of isoforms [14,15], which may be related to variable antimicrobial activities [14,15]. The peptide signals of crustin transcripts belonging to clusters 2, 3, and 4 are highly similar, as 14/19 amino acids share similar physico-chemical proprieties (Supplementary Figure S2), suggesting a similar secretory pathway regardless for these three crustin subfamilies.

### 3.2. Armadillidins, a New and Unique Crustacean AMP Family from Terrestrial Isopods

Armadillidin was first described and characterized in the terrestrial isopod species *A. vulgare* [10]. A variant peptide was later found in the same species with a Q at position 33 instead of a H [11]. To determine if these peptides are specific to *A. vulgare*, we screened the 21 transcriptome datasets. Our in silico analysis revealed that armadillidins are widespread in terrestrial isopod species, as full length sequences were found in 17 terrestrial isopod transcriptomes (Table 2). A single armadillidin sequence per transcriptome was identified in all datasets excepted in *Helleria brevicornis*. It is noteworthy that the identification of the armadillidin sequence of *Armadillo officinalis* was enabled by the similarity of its signal peptide with the other armadillidin sequences. The in silico search of armadillidins was extended to all crustacean transcriptome data available in NCBI, but all hits were from the terrestrial isopod transcriptomes from the study of [12], thereby confirming the specificity of these AMPs to Oniscidea species. This finding suggests that armadillidins evolved after the divergence between terrestrial isopods from other crustaceans, probably more than 100 million years ago [12].

**Figure 5:**
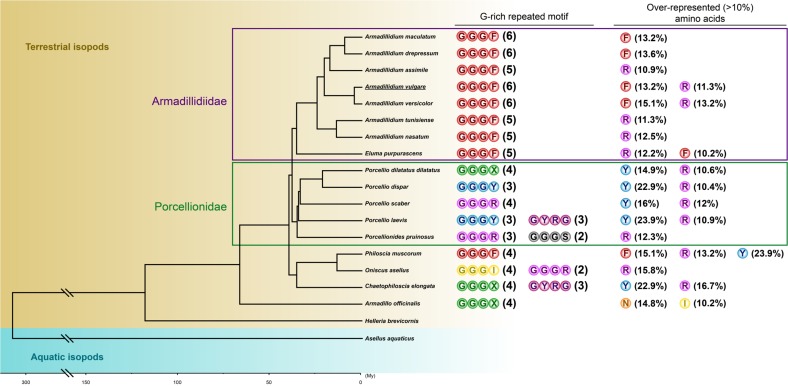
Structural characteristics (G-rich tetrapeptide repetitions and other overrepresented amino acids) of armadillidin sequences identified in terrestrial isopods. Phylogenetic relationships are based on [12]. Armadillidiidae and Porcellionidae families are framed in purple and green, respectively. The first armadillidin sequence was identified in *A. vulgare* (underlined). Overrepresented amino acids (>10% of the total length of mature sequence, other than G) are also indicated. Variable amino acids at the fourth position of the G-rich tetrapeptideare noticed with X.

All armadillidin sequences show the same arrangements of G-rich motif (GGGX), with variations concerning the number of repeats in the mature peptide sequence and the nature of the fourth amino acid composing this tetrapeptide pattern. On average, armadillidin peptides are composed of 5.1 (±1.0) G-rich motifs, ranging from 3 in *Porcellio dispar* to 7 in *Chaetophiloscia elongata* (Figure 5). Depending on the species, the fourth amino acid composing the G-rich tetrapeptide is F, Y, R, S, or I (Figure 5). Interestingly, the GGGF motif is systematically present in five or six copies in nine species (Table 2), including the entire Armadillidiidae family (comprising species of the genera *Armadillidium* and *Eluma*). This high conservation degree allows the definition of the following consensus sequence for armadillidins in the Armadillidiidae family: ----RPYIGGGG----GGG---GGGF--GGGF--GGGF-RGGG----G-----. This motif is suspected to be a key structural feature of those armadillidins and raises the question of its importance in the antimicrobial activity (Figure 5).

**Figure 6:**
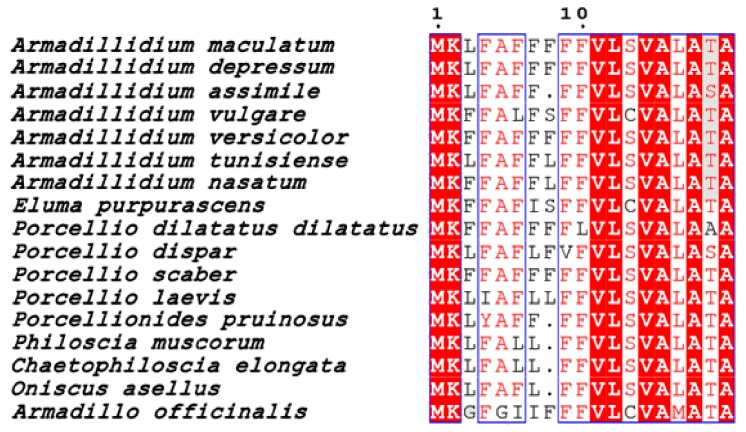
Multiple alignment of armadillidin signal peptide sequences. Amino acids highlighted in red are identical, those sharing the same physico-chemical properties are written in red, and blocks of aligned sequences sharing at least 70% of amino acid similarity are squared in blue.

More variability was observed among species of the family Porcellionidae, as all five species showed different G-rich repeated motifs (Figure 5). High variability was also recorded for armadillidins found in *Philoscia muscorum*, *Oniscus asellus*, *Chaetophiloscia elongata,* and *Armadillo officinalis* (Figure 5). It is noteworthy that arginine (R) is the main over-represented amino acid after G, as it represents >10% of the total length of mature armadillidins in 14 out of the 17 sequences identified (Figure 5). Another striking feature about armadillidins was the presence at the N-terminus of a highly conserved putative peptide signal sequence of 19 amino acids (Figure 6). This sequence is enriched in two amino acids, alanine (A) and phenylalanine (F). A BLAST search using the putative signal sequence of armadillidin H as a query against the nr/nt database shows armadillidin H as the only hit. Taken together, these results support that armadillidins are a new AMP family specifically from terrestrial isopods and with a markedly conserved signal sequence, which could be related to an unknown dedicated secretion system. This conservation degree was also found in other AMP families such as cathelicidins and dermaseptins, making the C-terminus part of peptides the region of greatest diversification that retains biological activities [29,30].

With the aim to determine if other members of the armadillidin family possess similar antibacterial activities to armadillidin H, two different peptides (armadillidin CE and armadillidin PP) were synthetized and tested against selected strains. These two peptides were selected on the basis of (i) the large sequence difference (armadillidin PP) from the original *A. vulgare* armadillidin (ii) the phylogenetic divergence (armadillidin CE) with *A. vulgare*. For each strain, minimum inhibitory concentrations (MICs) were determined and values were compared to those described in our previous study [11] (Table 3). Armadillidin CE was found to be poorly active against Gram-negative bacteria. This result is in agreement with previous findings on other glycine-rich AMPs like acanthoscurrins [31], ctenidins [32], holotricin 3 [33], leptoglycin [34], tenecin 3 [35], and shepherins [36]. Among sensitive strains, one was highly affected, *Micrococcus lysodeikticus*, as it was inhibited at low peptide concentration (4.75 µM; Table 1). Similar results were obtained for armadillidin PP, as determined MICs were in the same range as for armadillidin CE (Table 3). It is noteworthy that solubility of armadillidin CE was weaker in water/8% acetonitrile than armadillidin PP or armadillidin H and concentrations used in our assays were thus lower. In comparison, armadillidin H was previously described to be active against all tested bacteria in Table 1, except for two staphylococcal strains. Interestingly, *Bacillus megaterium* F04 and *Pseudomonas syringae* DC3000 were also highly sensitive to armadillidin H, contrary to both armadillidin CE and armadillidin PP in our conditions. Altogether, these results indicated that armadillidins display a quite similar wide antibacterial spectrum but with particular features depending on the tested peptide.

## 4. Conclusions

Our study revealed that terrestrial isopods, like all crustaceans studied to date, possessed an arsenal of AMPs composed of: (1) peptides common to all crustaceans or even arthropods (ALFs and crustins), and (2) a set of specific peptides, namely the armadillidin family. To date, no secondary structure could be defined for armadillidins probably because of their high G content, as already shown for Armadillidin H, nevertheless, the role of repeated G-rich motif (number of repeats, nature of the fourth amino acid) in the antimicrobial activity has to be addressed; further studies will thus be necessary to better grasp the structure-function relationship of all the family members. To improve our understanding of the biological roles played by armadillidins, it is necessary to characterize this new family in terms of in vivo biological activities, mode of action, tissue distribution and co-localization with other AMP members or other immunologic effectors. All these research efforts will also allow us to obtain a better integrated view of the role of armadillidins in host-microbiota interactions.

## Figures and Tables

**Table 1:** Classification of crustin transcripts identified in terrestrial isopod transcriptomes. The classification proposed by Vargas-Albores and Porchas [28] is based on the three main characteristics of the G-rich region: size, proportion of G content, and repetitive tetrapeptides occurring within the Gly-rich fragment. The classification proposed by [14] is based on the presence of a cysteine-rich domain, a glycine-rich hydrophobic domain, a proline and arginine rich region, an aromatic amino acid-rich domain, and on the number of WAP domain.

Crustin Cluster	Parameter	Mean ± Std	Range	Type (According to Vargas-Albores and Porchas, 2017)	Type (According to Tassanakajon et al., 2015)
1	Size	153.3 ± 15.8	130–166	II	II
	Glycine content (%)	30.1 ± 2.3	24.6–32.3		
	Repetitive Tetrapeptide	8.5 ± 2.2	3–11		
2	Size	38.8 ± 1.4	38–41	I	I
	Glycine content (%)	8.0 ± 0.7	7.3–9.8		
	Repetitive Tetrapeptide	0.0 ± 0.0	0–0		
3	Size	61.9 ± 0.2	61–62	I	I
	Glycine content (%)	7.2 ± 1.2	6.5–9.8		
	Repetitive Tetrapeptide	0.0 ± 0.0	0–0		
4	Size	39.2 ± 1.6	37–41	I	I
	Glycine content (%)	4.8 ± 2.5	2.5–9.3		
	Repetitive Tetrapeptide	0.0 ± 0.0	0–0		

**Table 2:** Sequences and structural parameters of armadillidins.

Organism	Mature Peptide Sequence	Length (aa)	Molecular Weight ^1^ (Da)	Glycine (%)	pI ^1^
*A. assimile*	GHIRRPYIGGGGLYGGGGGFHRGGGFHRGGGGFIGGGGFHRGGGFNRGGSYGYNG	55	5354.8	50.9	11.4
*A. depressum*	GGFGRPYIGGGGFNRGGGLHRGGGFNSGGGFNRGGGFNRGGGFNRGGGFHRGGSFGYNG	59	5726.1	49.2	12.1
*A. maculatum*	GGFGRPYIGGGGFNRGGGFHRGGGFRSGGGFHRGGGFNRGGGFHRGGSYGYNG	53	5252.6	49.1	11.9
*A. nasatum*	GHIGRPYIGGGGGGIYRGGGFRTGGGFRTGGGFHRGGGGFHRGGGFHRGGSYGYNG	56	5485.9	50	11.6
*A. tunisiense*	GHIGRPYIGGGGIYRGGGFRTGGGFHRGGGFHRGGGFQRGGGFYGGGSYGYNG	53	5268.7	49.1	11
*A. versicolor*	GGFGRPYIGGGGFNRGGGFHRGGGFNRGGGFHRGGGFNRGGGFHRGGSFGYNG	53	5263.6	49.1	12.1
*A. vulgare*	GHLGRPYIGGGGGFNRGGGFHRGGGFHRGGGFHSGGGFHRGGGFHSGGSFGYR	53	5259.6	47.2	12
	GHLGRPYIGGGGGFNRGGGFHRGGGFHRGGGFQSGGGFHRGGGFHSGGSFGYR	53	5250.6	47.2	12
*A. officinalis*	TFKPCGRSSGGSRCNRGYNRGIIGISGGNNKINGGGDFDDDDYESDYEDYNNGIIGIRGGTNTVNGGGSNNPKDSALKDYNNGIIGIGGGTNTVNGGGSNNPKDSAFKDPRGNRGIIGISGGRNVVQRG	129	13,190.1	24.8	8.6
*C. elongata*	TPGRPYYGGGYNGGYRGGYRRGGGFYGGGRFYGGGEGYRGGYYRGYRG	48	5150.5	45.8	10.1
*E. purpurascens*	SYVRRPYIGGGGGGFHRGGGFHRGGGFISGGGFHRGGGFNRGGGYGYNG	49	4892.3	46.9	11.4
*P. dilatatusdilatatus*	GHHGYGGSYGGRRYGHGGGRFGGIRGGGYGGGGHIGGGYGGYGGYRG	47	4490.7	57.4	10.1
*P. dispar*	GYIRKPYIGRGYGGGGYHRGGGFGYGGGYYRGGVGYGGGGYGGYGYRG	48	4855.2	52.1	9.8
*P. laevis*	SFIRKPYIGGGYGGYRGGGGYGGYRGGYYRGGGHYGGGYGGYGYRG	46	4729.1	50	9.8
*P. muscorum*	TFGRPYYGGGFNRGFGGGYHRGGGFHRGGGFYGGGFRGGYNRGYLG	46	4829.2	45.7	10.6
*P. pruinosus*	SYGRGSYGGGSIGRGSFGHGGGSFGRGGGRFGHGGGRFGGIGGGGRYGGGHIGGYRG	57	5307.6	54.4	11.6
*P. scaber*	GYIRRPVGYYGGGGGRYGGGRFGGGGGGIGGGRYGGGGRYGGGSYGGYHG	50	4709.0	58.0	10.2
*O. asellus*	TYRPSYGGGGGFNRGGGRGGGIHRGGGIGGGIYRGGGIGGGHRGGGGGRFNRGYGYR	57	5485.9	52.6	11.7

^1^ The average molecular weight and the theoretical pI were calculated using the online ProtParam tool (https://web.expasy.org/protparam/).

**Table 3:** Minimum inhibitory concentration (MIC) of armadillidin CE and PP against some bacterial strains.

		MIC of CE (µM)	MIC of PP (µM)	MIC of Armadillidin H (µM) (Verdon et al., 2016)
**Gram-positive bacteria**	*Bacillus megaterium* F04	>9.5	>19	2.37
	*Bacillus pumilus* NG1	9.5	19	4.75
	*Micrococcus lysodeikticus* ATCC 4698	4.75	4.75	2.37
	*Staphylococcus aureus* ATCC 29213	>9.5	>19	>19
	*Staphylococcus warneri* RK	>9.5	>19	>19
**Gram-negative bacteria**	*Escherichia coli* LMG 2092	>9.5	19	9.5
	*Klebsiella pneumoniae* 0502083	>9.5	19	19
	*Pseudomonas aeruginosa* PA14	>9.5	>19	19
	*Pseudomonas fluorescens* MFE01	>9.5	>19	19
	*Pseudomonas syringae* pv *tomato* DC3000	>9.5	>19	4.75

Bacteria (10^6^ CFU/mL) were incubated with two-fold dilutions of a 380 µM (PP) or 190 µM (CE) peptide stock solutions. Results correspond to the MIC after incubation for 24 h (bacteria) at 28 or 37 °C depending on the tested strain. Results are the mean of three independent experiments. Microbial strains were obtained from various culture collections: ATCC (American Type Culture Collection, LGC Standards, Molsheim, France) and LMG (Library of Microbiology Universiteit Gent, Gent, Belgium). Other strains were from the laboratory culture collection.

## Supplementary Materials

The following are available online at https://www.mdpi.com/2073-4425/11/1/93/s1. Supplementary File S1: Fasta file alignment of the ALF sequences used for the phylogenetic analysis. Supplementary File S2: Fasta file alignment of the crustin sequences used for the phylogenetic analysis. Supplementary File S3: Newick tree file generated after the phylogenetic analysis of the ALF transcripts. Supplementary File S4: Newick tree file generated after the phylogenetic analysis of the crustin transcripts. Supplementary Figure S1: Multiple alignment of the signal peptide sequences of ALFs clusterized after the phylogenetic analysis. Supplementary Figure S2: Multiple alignment of the signal peptide sequences of crustin clusterized after the phylogenetic analysis. Supplementary Table S1: Primers used for armadillidins characterization. Supplementary Table S2: Number of ALF and crustin transcripts identified in the 21 transcriptome datasets of terrestrial isopods.
