# Supplementary material for: Shedding Light on the Antimicrobial Peptide Arsenal of Terrestrial Isopods: Focus on Armadillidins, a New Crustacean AMP Family
Source: Genes (Basel). 2020 Jan 14;11(1):93. doi: 10.3390/genes11010093 (PMC7017220; doi:10.3390/genes11010093)
Supplement: Supplementary file 1 [file genes-11-00093-s001.zip › SupFile/Supplementary_Figure_S1.pptx]

## Slide 1
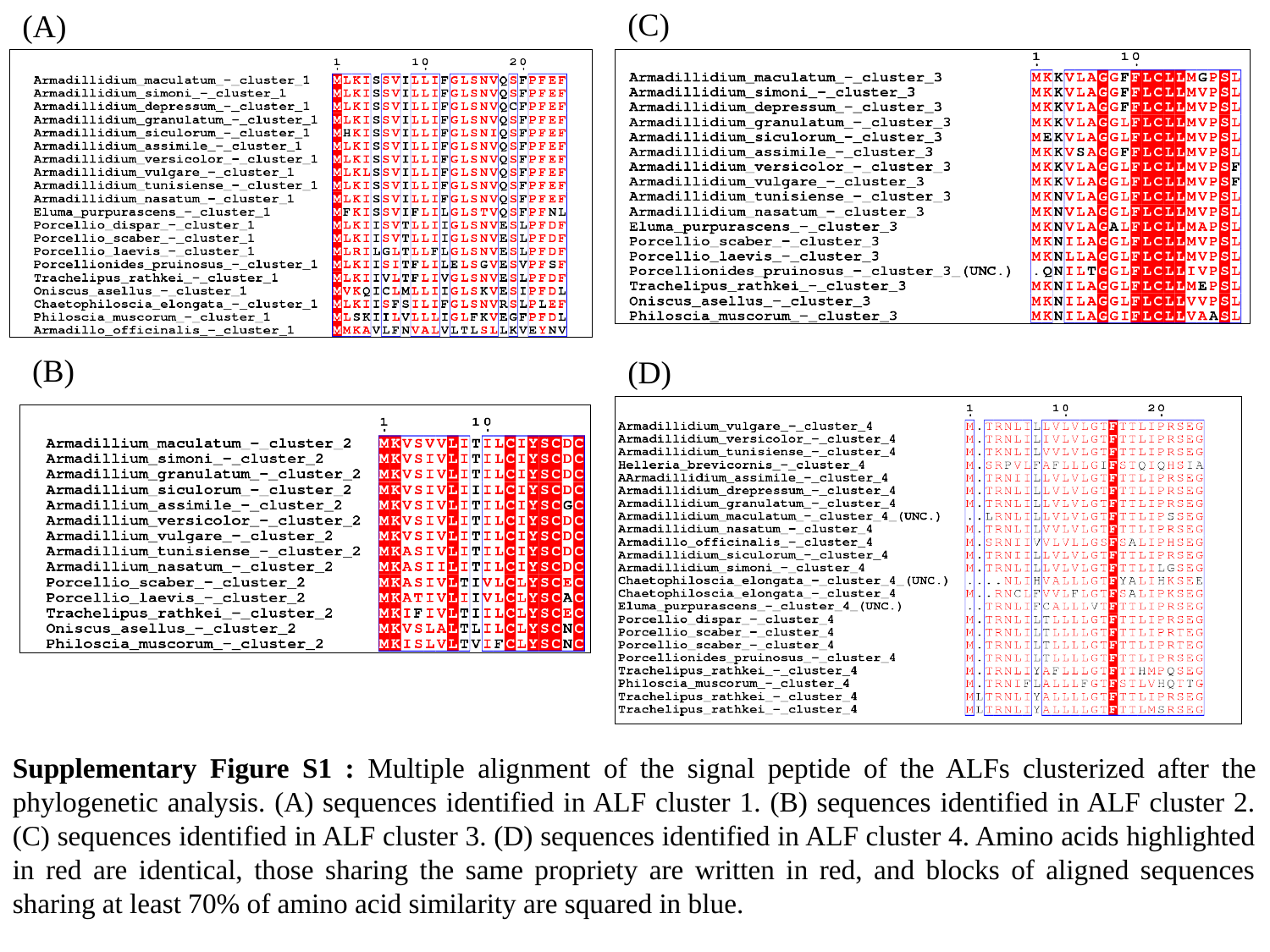

(C)
(A)
(B)
(D)
Supplementary Figure S1 : Multiple alignment of the signal peptide of the ALFs clusterized after the phylogenetic analysis. (A) sequences identified in ALF cluster 1. (B) sequences identified in ALF cluster 2. (C) sequences identified in ALF cluster 3. (D) sequences identified in ALF cluster 4. Amino acids highlighted in red are identical, those sharing the same propriety are written in red, and blocks of aligned sequences sharing at least 70% of amino acid similarity are squared in blue.
