# Supplementary material for: Shedding Light on the Antimicrobial Peptide Arsenal of Terrestrial Isopods: Focus on Armadillidins, a New Crustacean AMP Family
Source: Genes (Basel). 2020 Jan 14;11(1):93. doi: 10.3390/genes11010093 (PMC7017220; doi:10.3390/genes11010093)
Supplement: Supplementary file 1 [file genes-11-00093-s001.zip › SupFile/Supplementary_Figure_S2.pptx]

## Slide 1
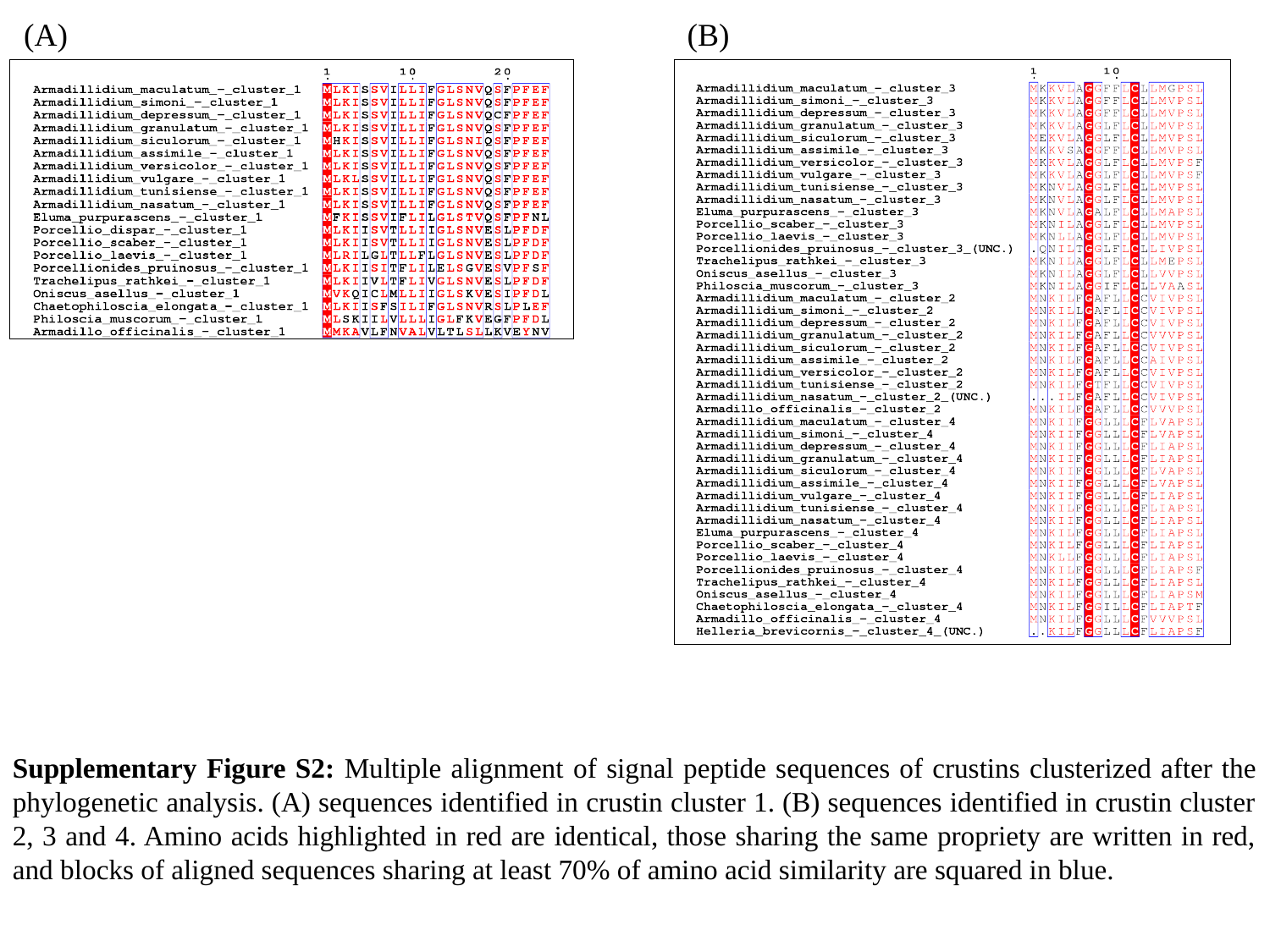

(A)
(B)
Supplementary Figure S2: Multiple alignment of signal peptide sequences of crustins clusterized after the phylogenetic analysis. (A) sequences identified in crustin cluster 1. (B) sequences identified in crustin cluster 2, 3 and 4. Amino acids highlighted in red are identical, those sharing the same propriety are written in red, and blocks of aligned sequences sharing at least 70% of amino acid similarity are squared in blue.
